# Quercetin attenuates inflammation in LPS‐induced lung epithelial cells via the Nrf2 signaling pathway

**DOI:** 10.1002/iid3.1185

**Published:** 2024-02-14

**Authors:** Pengju Lv, Pengli Han, Yuanbo Cui, Qiusheng Chen, Wei Cao

**Affiliations:** ^1^ Translational Medical Center Zhengzhou Central Hospital Affiliated to Zhengzhou University Zhengzhou China; ^2^ Department of Pulmonary and Critical Care Medicine Zhengzhou Central Hospital Affiliated to Zhengzhou University Zhengzhou China

**Keywords:** inflammatory cytokine, LPS, Nrf2, pneumonia, quercetin

## Abstract

**Background:**

Pneumonia is the leading cause of death among children under five, and kill almost two million children each year. Quercetin, a flavonoid polyphenolic compound, exerts many beneficial biological activities, including anti‐inflammatory functions. Our study aimed to investigate the possibility of quercetin as a therapeutic agent for pneumonia and its role in the inflammatory response induced by lipopolysaccharide (LPS).

**Methods:**

LPS induced human alveolar epithelial cell A549 as a lung inflammation model in vitro. The effects of quercetin on the production of cytokines and the expression of related‐proteins were detected by Enzyme‐Linked ImmunoSorbent Assay and Western Blot, respectively. Cell Counting Kit‐8 assay was used to detect cell viability. flow cytometry was used to measure cell apoptosis. NO levels were also analyzed through NO kit.

**Results:**

Our results found that quercetin attenuated the release of IL‐1β, IL‐6, PGE2, and nitrite in LPS‐induced A549 cells. In addition, quercetin inhibits cell apoptosis and relieves ROS generation in LPS‐induced A549 cells. Quercetin also inhibits LPS‐induced NF‐κB activation. They have upregulated the expression of nuclear factor erythroid 2 (Nrf2) and HO‐1.

**Conclusion:**

In conclusion, these results suggested that quercetin attenuates LPS‐induced inflammation in A549 by activating the Nrf2 signaling pathway.

## Graphical abstract

Our study aimed to investigate the possibility of quercetin as a therapeutic agent for pneumonia and its role in the inflammatory response induced by lipopolysaccharide (LPS)，In conclusion, we found that quercetin attenuates LPS‐induced inflammation in A549 by activating the nuclear factor erythroid 2 signaling pathway.

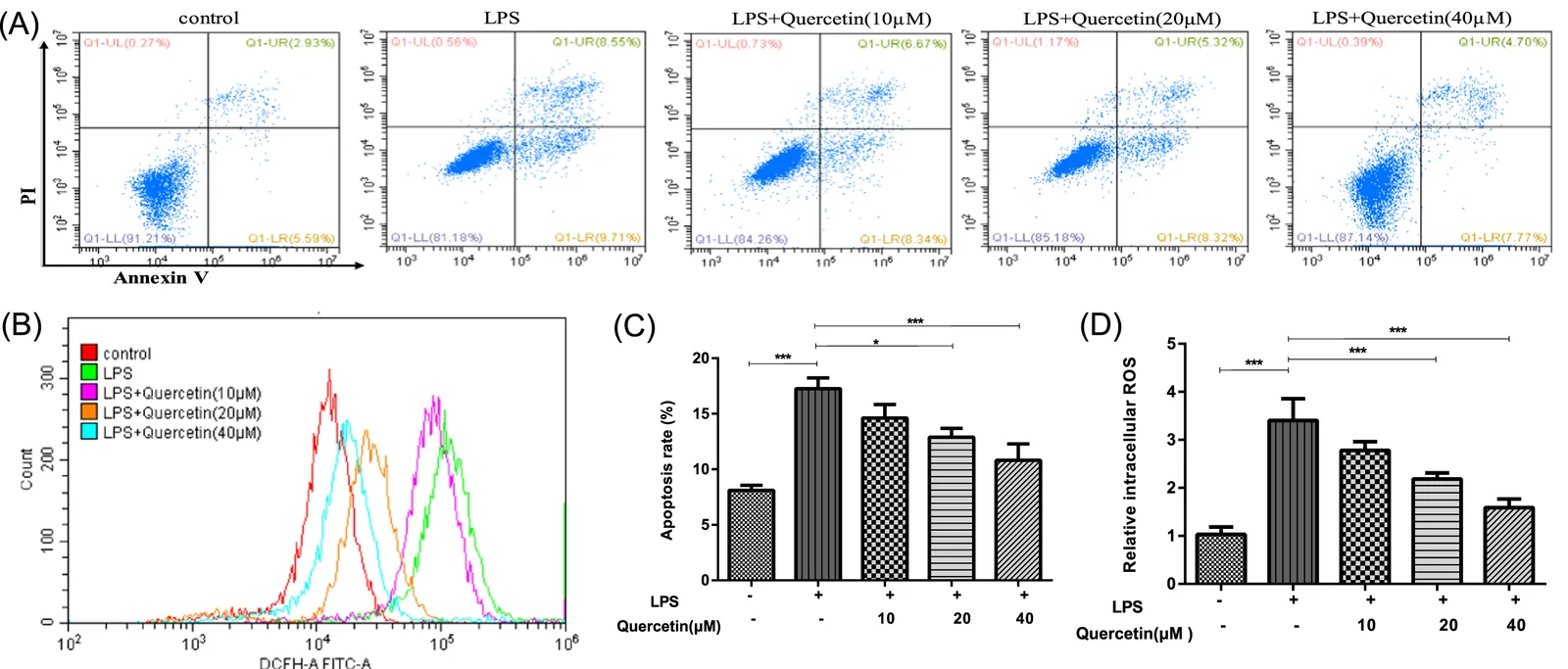

## 1 INTRODUCTION

Pneumonia is a class of inflammatory disease characterized by infection of pathogens such as bacteria, viruses, and fungi in lower respiratory tracts.^1^ Pneumonia remains a high mortality rate worldwide, especially in children and the elderly.^2^, ^3^ Up to now, pneumonia is mainly includes related to supportive therapy, antibiotics, oxygen, and assisted ventilation.^4^, ^5^ Nevertheless, the treatment effect is still unsatisfactory.^6^ Therefore, it is essential to identify the best targets for therapeutic intervention to alleviate pneumonia.

*Quercetin*, widely found in the bark, flower, leaf, bud, seed, and fruit of many plants, has a variety of bioactive flavonol compounds. Studies on the biological activity of quercetin have been known to have anticancer, antioxidant, anti‐inflammatory, and neuroprotective effects.^7^, ^8^, ^9^ Quercetin exerts gastroprotective activity against TNF‐α‐induced injury to the gastric mucosal epithelium.^10^ Quercetin regulates vascular endothelium function in chronic renal failure via modulation of Eph/Cav‐1 signaling.^11^ The antioxidant properties of quercetin have been widely reported in vivo and in vitro. Free radical scavenging activity of quercetin protects from various age‐associated disorders.^12^ Clinical studies show that quercetin supplementation prevents and treats chronic diseases such as hyperglycemia, inflammation, and hypertension.^13^ Quercetin has shown significant inhibitory effects on tumor progression via various mechanisms of action.^14^ However, the effects of quercetin on lipopolysaccharide (LPS)‐stimulated Type II alveolar cells and the underlying mechanism are still incompletely clear.

The nuclear factor erythroid 2‐related factor (Nrf2) is a redox‐sensitive transcription factor and plays a central role in inducing antioxidants to regulate related genes, which enhances cell survival.^15^ Therefore, the Nrf2 activity regulatory system has been proved to be an attractive drug target because of its complex mechanism. In addition, heme oxygenase (HO‐1), a downstream Nrf2 target protein, is an inducible isoform of HO‐1 and shows protective effects on pneumonocytes against lung inflammation.^16^, ^17^ Nrf2 and HO‐1 could potentially treat lung injury and diseases.

We consider direct intrapulmonary administration via the trachea with aerosolization for clinical application. Our study is based on Type II alveolar cell line A549 to investigate the anti‐inflammatory potency and mechanism of quercetin on LPS‐induced lung inflammation. The study could provide an important basis for quercetin treatment of lung inflammation.

## 2 MATERIALS AND METHODS

### 2.1 Chemicals and reagents

Quercetin (purity > 98%) and DMSO were obtained from Sigma, LPS and cell counting kit‐8 (CCK8) were purchased from Solarbio. The antibodies were obtained from Abcam. IL‐1β, IL‐6, NO, and PGE2 Enzyme‐Linked ImmunoSorbent Assay (ELISA) kits were purchased from Elabscience.

### 2.2 Cell culture and treatment

Human alveolar type II epithelial cells A549 was maintained in DMEM (Dulbecco's Modified Eagle Medium) supplemented with 10% fetal bovine serum at 37°C in an incubator of 5% CO_2_. LPS (10 μg/mL) was used to stimulate A549 cells for 24 an in vitro model for Pneumonia. Quercetin was then used to treat the cells for 24 h at indicated end concentrations after LPS stimulation. After that, the cell cultures were centrifuged at 3000 rpm for 10 min, and the supernatant was collected and divided into 500 µL per tube for ELISA assay and Nitrite determination. The cell extracts were harvested for Western blot analysis and ROS assay.

### 2.3 Cell viability

The cytotoxicity of quercetin on A549 was detected by the CCK8 assay. Briefly, A549 were seeded in 96‐well plates at a density of 3 × 10^5^ cells/well. After being treated with quercetin for 24 h. Ten micro liter CCK8 was added to each well and incubated for 3 h. the OD value was measured at 450 nm using a molecular device Microplate Reader (SpectraMax M2e).

### 2.4 Cell apoptosis

The cell apoptosis of A549 cells treated with quercetin was analyzed by flow cytometry. A549 cells were plated into a 12‐well plate at 2 × 10^6^ density for 24 h. after quercetin treatment, cells were quantified using an annexin V‐FITC apoptotic kit (7sea biotech). According to the manufacturer's protocol, the flow cytometry system (CytoFLEX; Beckman Coulter) was used to quantify apoptotic cells. Apoptotic rate of cells (number of apoptotic cells/total number of cells × 100%) was calculated.

### 2.5 ELISA assay

According to the manufacturer's instructions, the levels of IL‐1β, IL‐6, and PGE2 in the culture medium were detected by ELISA kits (Elabscience). A microplate reader (SpectraMax M2e) was used to examine the optical density at 450 nm.

### 2.6 Nitrite determination

As previously described, the nitrate concentration in the culture medium was measured using the Griess reagent assay.^18^ Briefly, 100 µL supernatant was mixed with 100 µL Griess reagent. Then, the absorbance was read at 525 nm using a spectrophotometer (SpectraMax M2e).

### 2.7 ROS assay

According to the manufacturer's protocol, the intracellular level of ROS was detected using a DCFDA cellular ROS assay kit (Solarbio). In brief, for the ROS detection of A549 cells, a total of 1.5 × 10^5^ cells were directly incubated with DCFDA solution(final concertration 15 μM) for 20 min at 37°C in an incubator of 5% CO_2_. The ROS level was detected in the flow cytometry system (CytoFLEX, Beckman Coulter).

### 2.8 Western blot analysis

According to the manufacturer's protocol, the total cell protein was extracted using RIPA Lysis Buffer (EpiZyme). Moreover, standard Western blot analysis techniques were utilized to analyze relative protein expression in cells. The bands were visualized using an ECL kit (EpiZyme). Expression levels were semi‐quantified relative to the optical density of β‐actin.

### 2.9 SiRNA transfection

For Nrf2 knockdown, si‐Nrf2 (5′‐GAC ATG GAT TTG ATT GAC ATA CT‐3′) were transfected into A549 cells using Lipofectamine 2000, transfection with siNC (5′‐CAC TTG AAT CCG ACG GAT TTG CA‐3′) as the control. Western blot was used to evaluate the knockdown efficiency.

### 2.10 Statistical analysis

The data presented are the means ± SEM of three independent experiments. Statistical analysis was performed with LSD's multiple comparison test. *p*＜.05 was considered to indicate a statistically significant difference. Comparisons between experimental groups were conducted using one‐way analysis of variances, whereas multiple comparisons were made using the LSD method. Statistical analyses were conducted using SPSS 19 (SPSS, Inc.)

## 3 RESULTS

### 3.1 Quercetin attenuates cellular damage of LPS‐induced A549 cells

**Figure 1:**
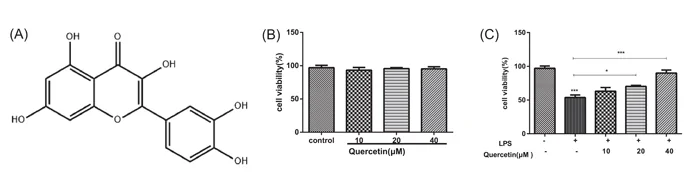
Quercetin attenuates cell damage of LPS‐induced A549 cells. (A) The chemical structure of quercetin. (B) the cell viability was detected by CCK8 assay after being treated with the different conditions of quercetin. (C) the cell viability was detected by CCK8 assay after being treated with the different conditions of quercetin in LPS‐induced cells. Data are shown as mean ± SEM (*n* = 3 independent experiments). Statistics: one‐way analysis of variances with Tukey's multiple comparison correction. *p < .05 and ***p < .001. compared to the control group.

The chemical structure of quercetin is shown in Figure 1A. After different concentrations (0, 10, 20, and 40 μM), quercetin was treated to A549 cells, and cell viability was measured by CCK8 assay. The data was shown in Figure 1B, which indicated no effect on the cell viability within this concentration range. After 10 μg/mL LPS induced A549 cells for 24 h, quercetin with different concentrations was treated to A549 cells. The results showed that the viability of A549 cells induced by LPS was increased significantly with the increased concentration of quercetin (Figure 1C). The results showed that quercetin could enhance the viability of LPS‐induced A549 cells.

### 3.2 Quercetin decrease the level of inflammatory factors released by LPS‐Induced A549 cells

**Figure 2:**
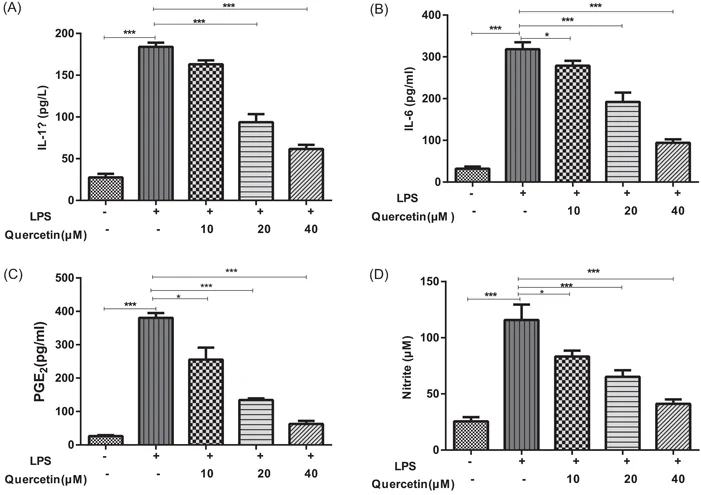
Quercetin inhibits inflammation of LPS‐induced A549 cells. a secreted level of IL‐1β (A), IL‐6 (B), PGE2 (C), and nitrite (D) ELISA assay detected in cultured cells. Data are shown as mean ± SEM (*n* = 3 independent experiments). Statistics: one‐way analysis of variances with Tukey's multiple comparison correction. *p < .05 and ***p < .001. compared to the control group.

To examine the inhibitory effects of quercetin on inflammatory mediators production, We detected the expression of cytokines IL‐1β (Figure 2A), IL‐6 (Figure 2B), and Nitrite (Figure 2C), PGE2 (Figure 2D) in A549 cells. Compared with the control group, the expression of inflammatory cytokines increased significantly in LPS‐induced A549 cells and dramatically decreased after quercetin treatment (Figure 2).

### 3.3 Quercetin inhibits cell apoptosis and ROS release in LPS‐Induced A549 cells

**Figure 3:**
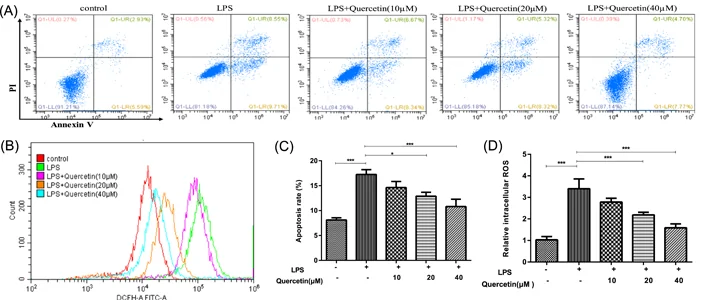
Quercetin Inhibits Cell Apoptosis and cellular ROS level in LPS‐Induced A549 Cells (A) the apoptosis rate of cultured A549 cells detected by Flow cytometry. (B) the apoptosis rate of LPS‐Induced A549 Cells was detected by Flow cytometry. (C) The apoptotic rate was quantified after LPS induction and gradient Quercetin treatment (D) Relative levels of intracellular ROS in A549 cells. *p < .05 and ***p < .001. The data presented are the means ± SEM of three independent experiments.

We used A549 epithelial cells to investigate the effect of quercetin on protecting lung tissues from LPS‐induced cell apoptosis and intracellular ROS increase. The data showed that after LPS treatment, the apoptotic rate remarkably elevated, while quercetin treatment decreased cell apoptosis (Figures 3A,C). Further, the level of intracellular ROS was quantified by the fluorescent probe DCFH‐DA. Consistent with the apoptotic assay, the intracellular ROS level increased by LPS remarkably, suppressed by quercetin treatment (Figures 3B,D). The data suggested that quercetin could protect cells from LPS‐induced ROS to prevent cell death.

### 3.4 Quercetin inhibits NF‐κB activation and induces Nrf2 activation in LPS‐induced A549 cells

**Figure 4:**
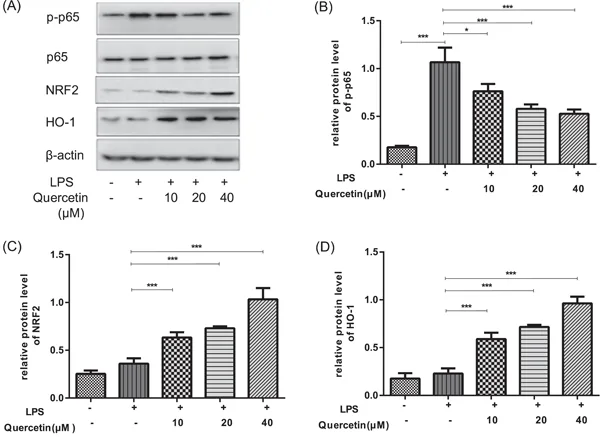
Quercetin inhibits NF‐κB activation and induces Nrf2 activation in LPS‐induced A549 cells. (A) images for p‐p65, Nrf2, and HO‐1 proteins expression in cultured cells after quercetin treatment. (B−D) Quantification of the relative expression level of p‐p65, Nrf2, and HO‐1. *p < .05 and ***p < .001. The data presented are the means ± SEM of three independent experiments.

To further investigate the anti‐inflammatory mechanism of quercetin on lung epithelial cells, we detected the effects of quercetin on NF‐κB activation in LPS‐induced A549 cells. The phosphorylation levels of NF‐κB p65 significantly increased in LPS‐induced A549 cells, compared with the LPS group, quercetin significantly inhibited NF‐κB activation induced by LPS(Figures 4A,B). and the expression of Nrf2 and HO‐1 were upregulated by quercetin, as shown in Figure 4A,C,D.

### 3.5 Quercetin attenuates LPS‐induced lung inflammation via Nrf2‐dependent activation

**Figure 5:**
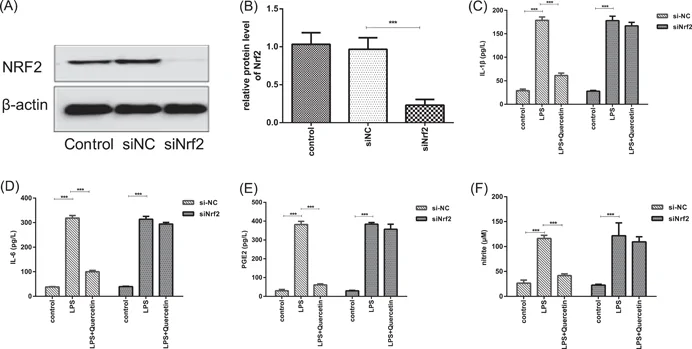
The anti‐inflammatory effects of quercetin were Nrf2 dependent. (A) Representative images for the expression of Nrf2 proteins in transfected cells. (B) Quantification of the relative expression level of Nrf2. ELISA assay detects a secreted level of IL‐1β (C), IL‐6 (D), PGE2(E), and nitrite (F) in cultured A549 cells. Data are shown as mean ± SEM (*n* = 3 independent experiments). Statistics: one‐way analysis of variances with Tukey's multiple comparison correction. *p < .05 and ***p < .001. compared to the control group.

To further confirm that the anti‐inflammatory mechanism of quercetin is Nrf2‐dependent, siRNA targeting Nrf2 was transfected. We also quantified the release of inflammatory cytokines from transfected‐A549 cells. ELISA assay showed the inhibition of quercetin on LPS‐induced IL‐1β, NO, IL‐6, and PGE2 in A549 were reversed when Nrf2 was knocked down (Figure 5).

## 4 DISCUSSION

This study discussed the protection of LPS‐induced lung inflammation by quercetin using A549 cell culture as pneumonia in vitro models. We tested whether quercetin could inhibit LPS‐induced inflammatory response in A549 cells. Our data showed that quercetin inhibited LPS‐induced inflammatory cytokines in A549 concentration‐dependent. Quercetin might be a candidate compound for the treatment of pneumonia.

Pneumonia is a form of acute respiratory infection that affects the lungs.^19^ Many studies have reported the critical role of inflammation in the progression of pneumonia.^20^ Nuclear factor‐kappa B is a dimeric transcription factor involved in host immune response, inflammation, cell proliferation, cell adhesion, growth signals, apoptosis defense, and cell differentiation. NF‐κB act as a principal component for pneumonia, asthma, COPD, and other inflammatory lung diseases.^21^ To investigate the anti‐inflammatory mechanism of quercetin, we detected the effect of quercetin on LPS‐induced NF‐κB activation and inflammation cytokines. Our results showed that quercetin suppresses the release of inflammatory mediators IL‐1β, IL‐6, PGE2, and nitrite in LPS‐induced A549 cells and attenuates LPS‐induced inflammation by inhibiting inflammatory cytokines production by blocking NF‐κB activity. Therefore, it might be a viable therapeutic strategy for treating pneumonia by inhibiting the NF‐κB activation.

In vivo studies have shown that Nrf2 plays a vital role in inflammatory diseases. Nrf2−/− animals showed more severe inflammation and tissue damage symptoms than WT animals,^22^, ^23^, ^24^ which indicated that Nrf2 negatively regulates the NF‐kB signaling pathway. Previous studies showed many natural herbal compounds with antioxidative and anti‐inflammatory effects by suppressing NF‐κB and activating Nrf2.^25^, ^26^, ^27^, ^28^ To investigate the regulatory mechanisms of quercetin on lung inflammation, we estimated the activities of Nrf2 in the present study. Our results showed that the treatment of quercetin upregulated the expression of Nrf2 and HO‐1. Furthermore, the anti‐inflammatory effects of quercetin were suppressed after the knockdown of Nrf2. The data indicated that quercetin inhibited LPS‐induced inflammatory response by activating the Nrf2 signaling pathway.

Our study found that quercetin significantly suppressed LPS‐induced IL‐1β, IL‐6, PGE2, and nitrite production in LPS‐induced pulmonary epithelial cells A549. The anti‐inflammatory mechanism of quercetin is due to its ability to suppress NF‐κB and activate Nrf2. These results indicated that quercetin might be a potential candidate for treating pneumonia.

Although our study show that the mechanism of attenuating pneumonia by quercetin, there are still some limination, we didn't asses the optimal dosing of the agent, which may impact the clinical translation of quercetin. second，in vivo experiment were not carried out in our study, limiting our ability to more accurately understand the detailed metabolic status of quercetin in vivo. This provides a meaningful focus for future research and we plan to address some of these issues in our upcoming work.

## AUTHOR CONTRIBUTIONS

Designed and performed the experiments: Pengju Lv, Pengli Han, Qiusheng Chen, Wei Cao. Statistical analysis: Pengju Lv,Yuanbo Cui. Writing—original draft: All authors. All authors read and approved the final manuscript.
